# On Using Linear Diophantine Equations for in-Parallel Hiding of Decision Tree Rules

**DOI:** 10.3390/e21010066

**Published:** 2019-01-14

**Authors:** Georgios Feretzakis, Dimitris Kalles, Vassilios S. Verykios

**Affiliations:** School of Science and Technology, Hellenic Open University, Patras 263 35, Greece

**Keywords:** decision trees, privacy preserving, Diophantine equations, hiding rules, entropy, information gain, data sharing

## Abstract

Data sharing among organizations has become an increasingly common procedure in several areas such as advertising, marketing, electronic commerce, banking, and insurance sectors. However, any organization will most likely try to keep some patterns as hidden as possible once it shares its datasets with others. This paper focuses on preserving the privacy of sensitive patterns when inducing decision trees. We adopt a record augmentation approach to hide critical classification rules in binary datasets. Such a hiding methodology is preferred over other heuristic solutions like output perturbation or cryptographic techniques, which limit the usability of the data, since the raw data itself is readily available for public use. We propose a look ahead technique using linear Diophantine equations to add the appropriate number of instances while maintaining the initial entropy of the nodes. This method can be used to hide one or more decision tree rules optimally.

## 1. Introduction

Privacy preserving data mining [1] is a relatively recent research area aimed at alleviating issues related to the use of data mining algorithms and the privacy of data subjects and the information or knowledge hidden in those data piles. Agrawal and Srikant [2] were the first to consider the induction of decision trees from anonymized data that were adequately corrupted with noise to survive from privacy attacks. The general strand of knowledge hiding research [3] has led to specific algorithms for hiding classification rules, such as the addition of noise, for example, by a data swap process [4].

A key element of privacy preserving data mining concerns individual data privacy and aims to shield the individual integrity of database records in order to prevent the re-identification of individuals or characteristic groups of data inference attacks. This forms the subject of this paper, which deals with the protection of sensitive patterns arising from the application of data mining techniques. Of course, all privacy preservation approaches strive to maintain data information quality.

The primary representative of statistical methods [5] adopts a parsimonious downgrading technique to determine whether additional confidentiality is worth the loss of functionality associated with not downgrading data. Reconstruction techniques involve the reconstruction of the public dataset [6,7] from the non-sensitive rules produced by C4.5 [8] and RIPPER [9] algorithms. Perturbation-based techniques involve the modification of transactions to only support non-sensitive rules [10], the removal of tuples associated with sensitive rules [11], the suppression of specific attribute values [12], and the redistribution of tuples supporting sensitive patterns to maintain the ordering of the rules [13]. Another interesting approach is machine learning classification over encrypted data [14], in which a private decision tree classifier allows the server to traverse a binary decision tree using the client’s input, such that the server does not learn the input x and the client does not learn the structure of the tree and the thresholds at each node. A recent work [15] proposes privacy-preserving decision tree evaluation protocols which hide the sensitive inputs from the counterparty using an additively homomorphic encryption (AHE), which are similar to the ElGamal encryption procedure.

In our previously published works [16,17], we proposed a series of techniques to adequately protect the disclosure of sensitive patterns of knowledge in the mining of classification rules. We aimed to hide sensitive rules without compromising the information value of the whole dataset. After an expert selected the sensitive rules, the class labels at the tree node corresponding to the tail of the sensitive pattern were modified (Swap-and-Add pass) to eliminate the gain achieved by the information metric that caused the split. By preserving the class balance of every node across the sensitive path (the path from the root until the leaf that we want to hide), we could assure that we would not have any change in the hierarchy order of the particular path due to changes in entropy of the nodes along the path. We then set the values of non-class attributes, appropriately adding new instances along the path to the root if necessary, so that non-sensitive patterns remained as unaffected as possible. This approach is very important because the sanitized dataset can be subsequently published and, even, shared with the dataset owner’s competitors, as can be the case with retail banking [18]. In this paper, we extend these works by formulating a generic look ahead technique that takes into account the structure of the decision tree all the way from an affected leaf to the root. The main contribution of this paper is to improve the Swap-and-Add pass by following a look ahead approach instead of a greedy approach, which was previously used. This technique can be accomplished by using linear Diophantine equations and, importantly, can handle in parallel any number of hiding requests by determining the overall minimum amount of added instances.

The rest of this paper consists of three sections: Section 2 describes the dataset operations we employ to hide a rule while attempting to affect the decision tree minimally; in Section 3 we present how the new look ahead approach can be applied in parallel hiding requests; and Section 4 discusses further research issues and concludes the paper.

## 2. Materials and Methods

We chose decision trees for our research since we are primarily interested in techniques that are applicable to "comprehensible" models, and this eventually leads to rules, trees, and other graphical models (Bayesian nets for example). However, the interpretability of rules and trees has to do with how widespread they are and with the scope to associate the masking of metric quality both regarding verboseness as well as the impact on understanding and the accuracy of concealment.

The transparency of the decision tree model is one of its great advantages. Unlike other decision-making models, the decision tree explicitly identifies all possible alternatives and traces each alternative in one view, allowing easy comparison between the different alternatives. One of the main benefits of decision tree analysis is its ability to assign specific values to the problems, decisions, and results of each decision. This reduces ambiguity related to decision-making. Every possible decision scenario finds representation through a clear fork and node, allowing all possible solutions to be seen in one view. A decision tree also allows data to be partitioned at a much deeper level, which is not easily achieved with other decision-making classifiers, such as logistic regression. The decision tree illustrates the problem graphically and various alternatives in a simple and easy-to-understand format that requires no explanation. Decision trees divide data into illustrations that are easy to understand based on rules easily understood. Simple mathematics based on entropy concepts can easily replicate the reasons for the rules produced in a decision tree.

**Figure 1:**
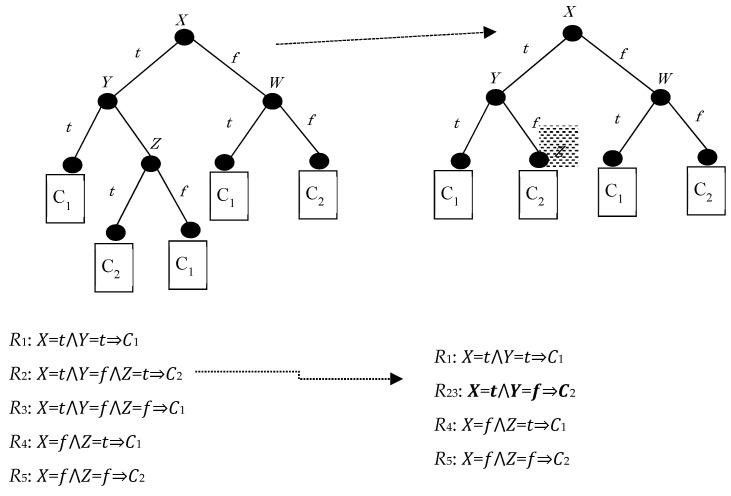
A binary decision tree, before (left) and after (right) hiding and the associated rule sets.

Figure 1 below shows a baseline problem, which assumes a binary decision tree representation, with binary-valued symbolic attributes (X, Y, and Z) and binary classes (C1 and C2). Hiding R3 implies that the splitting in node Z should be suppressed, hiding R2 as well.

A first approach to hide R3 would be to remove all the instances of the leaf corresponding to R3 from the training data and to retrain the tree from the resulting dataset. However, this action can lead to a significant restructuring of the tree which also affects other parts of the tree. 

Another approach would be to turn the direct parent of the R3 leaf into a new leaf. This would not, however, modify the actual dataset. An opponent could thus recover the original tree.

To achieve hiding by minimally modifying the original dataset, we may interpret “minimally” concerning dataset changes or whether the sanitized decision tree produced via hiding is syntactically close to the original. Measuring minimality in how one modifies decision trees has been studied regarding heuristics that guarantee or approximate the impact of changes [19,20,21].

Hiding in Z, however, changes the statistics along with the path from Z to the root. Since the splitting along this path depends on these statistics, the relative ranking of the attributes may change if we use the same induction algorithm on the modified dataset. To avoid ending with an entirely different tree, we first used a bottom-up pass (Swap-and-Add) to change the class label of instances at the leaves, and then added new instances on the root path to preserve the critical statistics on the intermediate nodes.

Subsequently, we use a top-down pass (Allocate-and-Set) to complete the newly added instance specification. These two passes help us firstly hide all sensitive rules and secondly keep the sanitized tree close to the structure of the original decision tree. We note that the above two procedures had been fully described in previously published works [16,17].

### 2.1. Adding Instances to Preserve the Class Balance Using Linear Diophantine Equations: A Proof of Concept and an Indicative Example

**Figure 2:**
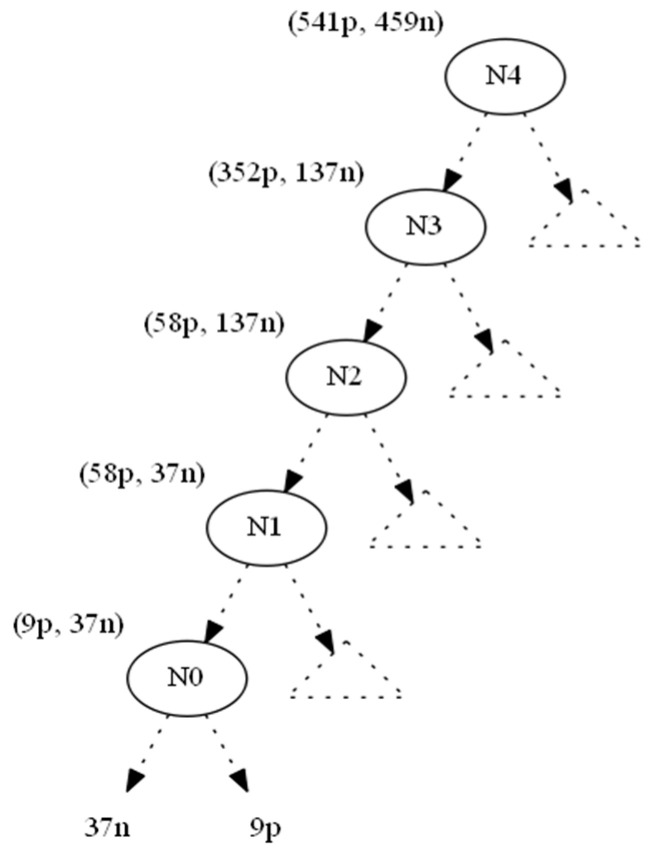
Original tree.

The Swap-and-Add pass aims to ensure that node statistics change without threatening class-value balances in the rest of the tree. Using Figure 2 as an example, we show the original tree with class distributions of instances across edges.

**Figure 3:**
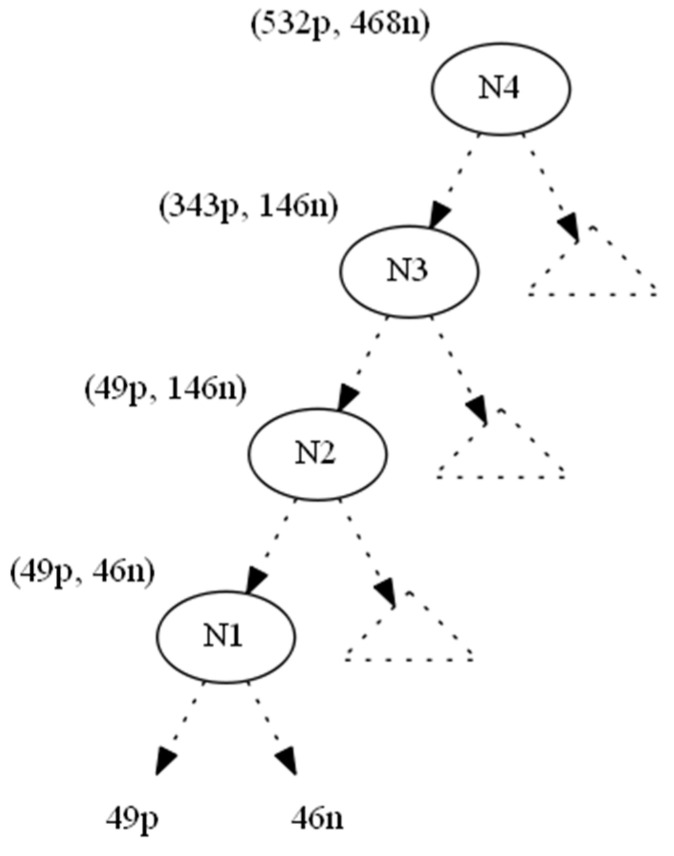
Bottom-up propagation of instances (−9p,+9n).

We used the information gain as the splitting heuristic. In order to hide the leaf which corresponds to the nine positive instances (to the right of N0), we changed the nine positive instances to negative ones and denoted this operation by (−9p,+9n). As a result, the parent node N0 becomes a one-class node with minimum (zero) entropy. All nodes located upwards to node N0 until the root N4 also absorb the (−9p, +9n) operation (Figure 3). This conversion would leave node Ν1 with 49p+46n instances. However, since its original 58p+37n distribution contributed to N1’s splitting attribute AN1, which in turn created N0 (and then 9p), we should preserve the information gain of AN1, since the entropy of a node depends only on the ratio p:n of its instance classes (Lemma 1).

**Lemma** **1.** *The entropy of a node only depends on the ratio of its instance classes.* (The proof is in Appendix A)

To maintain the initial ratio of node N1 (58p:37n), an appropriate number of positive and negative instances should be added to node N1, and this addition process should be extended to the tree root by accumulating all instance requests from below at each node and by adding instances to maintain the node statistics locally, propagating these changes to the root. 

In our previous published work [16,17], the above procedure was greedy, mostly solving the issue for only one (tree) level of nodes, which often resulted in a non-minimum number of added instances, whereas a look ahead based solution would be able to take into account all levels up to the root. Furthermore, the new ratios of the nodes (p:n) were not the same as they were before the change, thus propagating ratio changes, whose impact could only be quantified in a compound fashion by inspecting the final tree, hampering our ability to investigate the behaviour of this heuristic in a detailed fashion. Therefore, we used linear Diophantine equations as the formulation technique of the problem of determining how many instances to add; as we will show, this technique deals with both issues in one go.

**Definition** **1.** *A Diophantine equation is a polynomial equation where the coefficients are integers, and the solutions are integers. The most basic Diophantine equation is the linear case and is of the following form:*

*ax* + *by* = *c* *where a, b, c* ∈ ℤ

Referring to the example in Figure 3, let $\left(x_{1},y_{1}\right)$ be the number of positive and negative instances, respectively, that must be added to node N1 (49p,46n) in order to maintain its initial ratio (58p,37n), as shown in Figure 2. This can be expressed with the following equation:

$$\frac{49+x_{1}}{46+y_{1}}=\frac{58}{37}$$

The above equation is equivalent to the following linear Diophantine equation:

(1) $$37x_{1}-58y_{1}=85$$

Similarly, let ($x_{2},y_{2}),\left(x_{3},y_{3}\right),\left(x_{4},y_{4}\right)$ be the corresponding number of positive and negative instances that have to be added to nodes N2, N3 and N4.

The corresponding linear Diophantine equations for nodes N2, N3 and N4 are:

(2) $$137x_{2}-58y_{2}=1755$$

(3) $$137x_{3}-352y_{3}=4401$$

(4) $$459x_{4}-541y_{4}=9000$$

**Theorem** **1.** *Let a, b and c be integers with a and b, not both zero. The linear Diophantine equation*

*ax* + *by* = *c* *where a*, *b*, *c* ∈ ℤ

*has a solution if and only if* d=GCD(a,b) *divides c.*

If a linear Diophantine equation does not have solutions, this problem can be overcome by a slight change in the initial ratio until a solvable linear Diophantine equation is achieved.

**Theorem** **2.** *Let a and b integers with d=GCD(a,b). If* c mod d=0, *then there are infinite integer solutions. Moreover, if*$x=x_{0}\;,\;y=y_{0}$*is a particular solution of the equation, then all solutions are given by*

$$x=x_{0}+\frac{b}{d}n\;,\;y=y_{0}-\frac{a}{d}n\;\mathrm{where}\;n\in \mathbb{Z}$$

The general solutions of the above four (1–4) linear Diophantine equations are given below (k∈ℤ):

$$37x_{1}-58y_{1}=855\Leftrightarrow \{\begin{array}{c} x_{1}=9405+58k\\ y_{1}=5985+37k \end{array}\;$$

$$137x_{2}-58y_{2}=1755\Leftrightarrow \{\begin{array}{c} x_{2}=-19305+58k\\ y_{2}=-45630+137k \end{array}\;$$

$$137x_{3}-352y_{3}=4401\Leftrightarrow \{\begin{array}{c} x_{3}=-734967+352k\\ y_{3}=-286065+137k \end{array}\;$$

$$459x_{4}-541y_{4}=9000\Leftrightarrow \{\begin{array}{c} x_{4}=-297000+541k\\ y_{4}=-252000+459k \end{array}$$

From the infinite pairs of solutions for every linear Diophantine equation, we choose the pairs $\;\left(x_{1}^{*},y_{1}^{*}\right),\;\left(x_{2}^{*},y_{2}^{*}\right),\;\left(x_{3}^{*},y_{3}^{*}\right)\;\mathrm{and}\;\left(x_{4}^{*},y_{4}^{*}\right)$, where $x_{1}^{*},x_{2}^{*},x_{3}^{*},x_{4}^{*}$, $y_{1}^{*},y_{2}^{*},y_{3}^{*},\;y_{4}^{*}$ are the minimum natural numbers that satisfy the following condition.

(C1): $x_{1}^{*}\leq x_{2}^{*}\leq x_{3}^{*}\leq x_{4}^{*}$ and $y_{1}^{*}\leq y_{2}^{*}\leq y_{3}^{*}\leq y_{4}^{*}$.

Condition (C1) ensures that we have selected those pairs of solutions which have the minimum total sum of positive and negative instances that have to be added in each node, starting from the lowest node up to the root of the decision tree, given that every addition of instances propagates upwards in a consistent manner (i.e., if one adds some instances at a lower node, one cannot have added fewer instances in an ancestor node).

With this technique, we can determine precisely the minimum number of instances that must be added to each node to maintain the initial ratios of every node. 

For this example, the pairs of solutions that are both minimum and satisfy the condition (C1) are

$$\left(x_{1}^{*},y_{1}^{*}\right)=\left(67,28\right)\;,\left(x_{2}^{*},y_{2}^{*}\right)=\left(67,128\right),\left(x_{3}^{*},y_{3}^{*}\right)=\left(361,128\right),\left(x_{4}^{*},y_{4}^{*}\right)=\left(550,450\right)$$

Based on the above solutions, we must add 67 positive and 28 negative instances to N1, which leads to a ratio of $\left(\frac{116p}{74n}\right)$. These new instances propagate upwards; therefore, on N2 we do not need to add any positive instances, but we do need to add 100 (=128 − 28) negative instances, which leads to a ratio of $\left(\frac{116p}{274n}\right)$. Similarly, for N3 we should add 294 (=361 − 67) new positive and no negative instances. Finally, for N4 we should add 189 (=550 − 361) new positive and 322 (=450 − 128) new negative instances. With this look ahead technique, we know from the very beginning the minimum number of instances that we must add in order to maintain the exact values of initial ratios. 

We note that the solutions of Diophantine Equation (4), corresponding to the node N4 (root), determine the total number of instances that should be added to our dataset to maintain the same ratios. If we change the N4 ratio slightly (in our case let be changed to $\left(\frac{540p}{460n}\right)$ instead of $\left(\frac{541p}{459n}\right)$), then we have a different Diophantine equation that results in a smaller number of additional instances. In our example, the new Diophantine Equation (5) and the corresponding set of solutions are shown below:

(5) $$460x_{4}-540y_{4}=4000$$

$$460x_{4}-540y_{4}=4000\Leftrightarrow \{\begin{array}{c} x_{4}=-1400+27k\\ y_{4}=-1200+23k \end{array},\;k\in \mathbb{Z}$$

For this example, the pairs of solutions that are both minimum and satisfy the condition C1 are

$$\left(x_{1}^{*},y_{1}^{*}\right)=\left(67,28\right)\;,\left(x_{2}^{*},y_{2}^{*}\right)=\left(67,128\right),\left(x_{3}^{*},y_{3}^{*}\right)=\left(361,128\right),\left(x_{4}^{*},y_{4}^{*}\right)=\left(382,318\right)$$

Therefore, we have to add 700 new instances instead of 1000 that we had to add before. Of course, now we do not have the same ratio p:n for node N4, but something very close to it. In other words, the method of linear Diophantine equations helped us to make a holistic tree-wide trade-off between the number of added instances and the degree of precision to which we approximate the original node’s ratio.

### 2.2. Fully Specifying Instances

For the newly added instances, setting the values of some attributes is only a partial instance specification because we did not fix those instance values for any other attribute, other than those present in the path from the root to the node where the addition of the instances occurred. Unspecified values must be set in such a way to ensure that competing attributes do not displace currently selected attributes at all nodes; this is what the Allocate-and-Set pass does.

Concerning Figure 2 and the 9n instances added due to N1 via the N2-N1 branch, these instances did not have their values set for $A_{N1}$ and $A_{N2}$. These must be set accordingly to minimize the possibility that $A_{N2}$ is displaced from N2, since (at N2) any of the attributes $A_{N0}$, $A_{N1}$ or $A_{N2}$ (or any other) can be selected. Those 9n instances were added to help guarantee the existence of N1. 

As in the bottom-up pass, we needed the information gain of $A_{N2}$ to be sufficiently large to prevent competition from $A_{N0}$ or $A_{N1}$ at node N2, but not too large to threaten $A_{N3}$. We started with the best possible allocation of values to attribute $A_{N2}$, and gradually explored directing some values along the N2–N1 branch, stopping when the information gain for $\;A_{N2}$ was lower than the information gain for $A_{N3}$. We use the term two-level hold-back to refer to this technique because it covers two levels of the tree. This approach takes advantage of convexity property of the information gain difference function (Lemma 2).

The Allocate-and-Set pass examines all four distribution combinations of all positive and all negative instances to one branch, selecting the one that maximizes the information gain difference and then moving along the slope that reduces the information gain, until we do not exceed the parent’s information gain. Following this, the recursive specification was performed all the way to the tree fringe.

**Lemma** **2.** *Distributing new class instances along only one branch maximizes information gain.* (The proof is in Appendix A)

### 2.3. Hiding in Parallel: Grouping of Hiding Requests

By processing hiding requests serially, each entails the full cost of updating the instance population. By knowing them in advance, we only consider every node once in the bottom-up and once in the top-down pass. We express that dealing with all hiding requests in parallel leads to the minimum number of new instances by

$$\left|T_{R}^{P}\right|=\underset{i}{min}\left|{\left(T_{\left\{i\right\}}^{S}\right)}_{R-\left\{i\right\}}^{S}\right|$$

The formula states that for a tree *T*, after a parallel (*Tp*) hiding process of all rules (leaves) in *R*, the number of instances (|*T*|) is the optimum along all possible orders of all serial (*Ts*) hiding requests drawn from *R*. A serial hiding request is carried out by selecting a leaf to be hidden, after which the remaining leaves are treated recursively.

**Lemma** **3.** *When serially hiding two non-sibling leaves, the number of new instances to be added to maintain the max:min ratios is larger or equal to the number of instances that would have been added if the hiding requests were handled in parallel.* (The proof is in Appendix A)

## 3. Results

**Figure 4:**
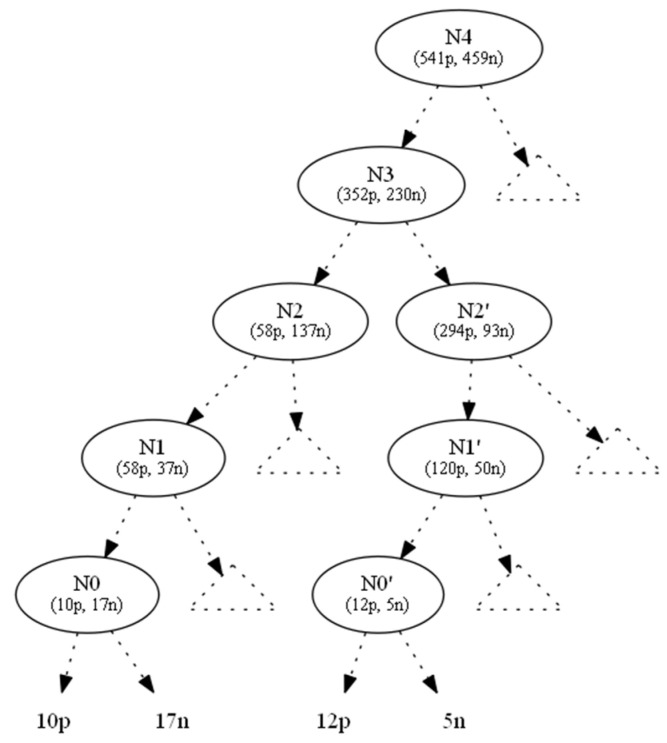
Original tree.

In this section we demonstrate an example in which two hiding requests were handled in parallel, using the proposed look ahead technique of linear Diophantine equations. In Figure 4, we show the original tree with class distributions on nodes.

**Figure 5:**
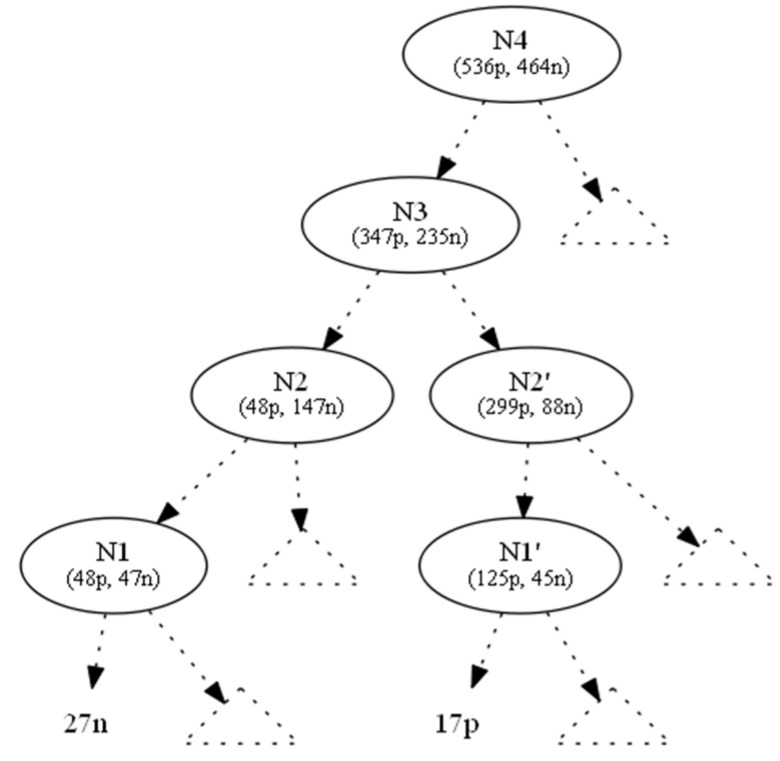
Bottom-up propagation of instances (−10p,+10n) from the left (+5p,−5n) and right side of the tree.

We used the information gain as the splitting heuristic. To hide the leaf which corresponds to the ten positive instances (to the left of N0), we changed the ten positive instances to negative ones and denoted this operation by (−10p, +10n). As a result, the parent node N0 became a one-class node with minimum (zero) entropy. All nodes located upwards of node N0 until the root N4 also absorbed the (−10p, +10n) operation (Figure 5). This conversion left Ν1 with 48p + 47n instances. But, as its initial 58p + 37n distribution contributed to N1’s splitting attribute, AN1, which in turn created N0 (and then 10p), we preserved the information gain of AN1, since the entropy of a node only depends on the ratio p:n of its instance classes. 

To hide the leaf which corresponds to the five negative instances (to the right of N0’), we changed the five negative instances to positive ones and denote this operation by (+5p, −5n). As a result, the parent node N0’ became a one-class node with minimum (zero) entropy. All nodes located upwards to node N0’ until the root N4 also absorbed the (−10p, +10n) operation (Figure 5). The intersection node N3 and the root N4 were affected by (−5p, +5n), which is the total outcome of the two operations from the two subtrees below N3. 

This conversion left Ν1’ with 125p + 45n instances. But, as its initial 120p + 50n distribution contributed to N1’s splitting attribute, AN1’, which in turn created N0’ (and then 5n), we preserved the information gain of AN1’, since the entropy of a node only depends on the ratio p:n of its instance classes.

In order to maintain the ratio of nodes N1 and N1’, we had to add an appropriate number of positive and negative instances to N1, N1’ and extend this addition process to the tree root, by accumulating at each node all instance requests from below and by adding instances locally to maintain the node statistics, propagating these changes to the tree root. 

Let $\left(x_{1},y_{1}\right)$ be the number of positive and negative instances, respectively, that should be added to node N1 to maintain its initial ratio. This can be expressed with the following equation:

$$\frac{48+x_{1}}{47+y_{1}}=\frac{58}{37}$$

The above equation is equivalent to the following linear Diophantine equation:

(6) $$37x_{1}-58y_{1}=950$$

Similarly, let ($x_{2},y_{2}),\;\left(x_{1}^{\prime },y_{1}^{\prime }\right),\left(x_{2}\prime ,y_{2}\prime \right),\left(x_{3},y_{3}\right),\;\left(x_{4},y_{4}\right)$ be the corresponding number of positive and negative instances that should be added to nodes N2, N1’, N2’, N3 and N4.

The corresponding linear Diophantine equations for nodes N2, N1’, N2’, N3 and N4 are:

(7) $$137x_{2}-58y_{2}=1950$$

(8) $$50x_{1}^{\prime }-120y_{1}^{\prime }=-850$$

(9) $$93x_{2}^{\prime }-294y_{2}^{\prime }=-1935$$

(10) $$230x_{3}-352y_{3}=2910$$

(11) $$459x_{4}-541y_{4}=5000$$

The general solutions of the above six (6–11) linear Diophantine equations are given below ( k∈ℤ):

$$37x_{1}-58y_{1}=950\Leftrightarrow \{\begin{array}{c} x_{1}=10450\;+\;58k\\ y_{1}=6650\;+\;37k \end{array}$$

$$137x_{2}-58y_{2}=1950\Leftrightarrow \{\begin{array}{c} x_{2}=-21450\;+\;58k\\ y_{2}=-50700\;+\;137k \end{array}\;50x_{1}^{\prime }-120y_{1}^{\prime }=-850\Leftrightarrow \{\begin{array}{c} x_{1}\prime =-425\;+\;12k\\ y_{1}\prime =-170\;+\;5k \end{array}\;137x_{2}\prime -58y_{2}\prime =1755\Leftrightarrow \{\begin{array}{c} x_{2}\prime =-12255\;+\;98k\\ y_{2}\prime =-3870\;+\;31k \end{array}\;137x_{3}-352y_{3}=4401\Leftrightarrow \{\begin{array}{c} x_{3}=109125\;+\;176k\\ y_{3}=71295\;+\;115k \end{array}\;459x_{4}-541y_{4}=9000\Leftrightarrow \{\begin{array}{c} x_{4}=-165000\;+\;541k\\ y_{4}=-140000\;+\;459k \end{array}$$

From the infinite pairs of solutions for every linear Diophantine equation, we choose the pairs $\;\left(\bar{x_{1}},\bar{y_{1}}\right),\left(\bar{x_{2}},\bar{y_{2}}\right),\left(\bar{x_{1}}\prime ,\bar{y_{1}}\prime \right),\left(\bar{x_{2}}\prime ,\bar{y_{2}}\prime \right),\;\left(\bar{x_{3}},\bar{y_{3}}\right),\;\left(\bar{x_{4}},\bar{y_{4}}\right)$ where $\;\bar{x_{1}},\bar{x_{2}},{\bar{x_{1}}}^{\prime },{\bar{x_{2}}}^{\prime },\bar{x_{3}},\bar{x_{4}},\bar{y_{1}},\bar{y_{2}},{\bar{y_{1}}}^{\prime },{\bar{y_{2}}}^{\prime },\bar{y_{3}},\bar{y_{4}}$ are the minimum natural numbers that satisfy the conditions (C1) and (C2).

(C1): $\bar{x_{1}}\leq \bar{x_{2}}$ and $\bar{y_{1}}\leq \bar{y_{2}}$ and ${\bar{x_{1}}}^{\prime }\leq {\bar{x_{2}}}^{\prime }$ and ${\bar{y_{1}}}^{\prime }\leq {\bar{y_{2}}}^{\prime }$

(C2): $\bar{x_{2}}+{\bar{x_{2}}}^{\prime }\leq \bar{x_{3}}\leq \bar{x_{4}}$ and $\bar{y_{2}}+{\bar{y_{2}}}^{\prime }\leq \bar{y_{3}}\leq \bar{y_{4}}$

Condition (C1) ensures that we have selected the optimum path from the leaves up to the intersection node N3 of the decision tree.

Condition (C2) ensures that we have selected the optimum path from one level below the intersection node N3 (N2, N2’) up to the root.

For this example, the pairs of solutions that are both minimum and satisfy the conditions (C1), (C2) are:

$$\left(\bar{x_{1}},\bar{y_{1}}\right)=\left(68,27\right)\;,\left(\bar{x_{2}},\bar{y_{2}}\right)=\left(68,127\right),\left(\bar{x_{1}}\prime ,\bar{y_{1}}\prime \right)=\left(7,10\right),\left(\bar{x_{2}}\prime ,\bar{y_{2}}\prime \right)=\left(93,36\right)$$

$$\left(\bar{x_{3}},\bar{y_{3}}\right)=\left(357,225\right)\;,\left(\bar{x_{4}},\bar{y_{4}}\right)=\left(546,454\right)$$

Based on the above solutions, we should add to N1 68 positive and 27 negative instances. These new instances propagate upwards; therefore, at N2 we did not need to add any positive instances, but we needed to add 100 (=127 − 27) negative instances. In the same manner, we needed to add to N1’ seven positive and ten negative instances. These new instances propagate upwards; therefore, on N2’ we needed to add 86 (=93 − 7) positive instances and 26 (=36 − 10) negative instances. 

Similarly, for N3, which is an intersection node, we should add the corresponding instances from its children (N2, N2’), 196 (=357 − 68 − 93) new positive and 62 (=225 − 127 − 36) new negative instances. Finally, for N4, we should add 189 (=546 − 357) new positive and 229 (=454 − 225) new negative instances. 

Based on this example, we observe that by using this technique we can handle more than one hiding request without any increase in the number of instances that need to be added. An algorithm that processes in parallel *n* hiding requests is described in detail in Appendix B. We have also developed a prototype to demonstrate the validity of our arguments on a small scale example [22]. This implementation demonstrates the use of Diophantine equations on our technique and consists of only one part of our proposed method. For that reason, there is no need at this stage to use real datasets.

## 4. Brief Discussion and Conclusions

We have presented a new look ahead technique for deciding how many instances to add to a decision tree to hide a specific rule. By using linear Diophantine equations instead of the previously used greedy approach, our heuristic allows one to specify which decision tree leaves should be hidden, and then intelligently add instances to the original dataset so that the next time one tries to build the decision tree (with the same induction algorithm), the to-be-hidden nodes will have disappeared, as the instances corresponding to these nodes will have been absorbed by their neighbors.

Two fully-fledged examples of the proposed approach have been presented: One for a single request and the other for two parallel hiding requests. Also, we have introduced an algorithm for n parallel hiding requests for this look ahead technique.

With regards to performance aspects, besides speed, the issue of assessing the similarity of the original tree to the one produced after the above procedure has been applied must also be considered. Another issue of substantial importance is syntactic similarity [23] (comparing the data structures—or parts thereof—themselves) or semantic similarity (comparing against reference datasets), which will also help settle questions of which heuristics work better and which not.

As the number of instances to be added is a primary index of the heuristic’s quality, a reasonable direction for investigation is to determine the appropriate ratio values, which result in smaller integer solutions of the corresponding linear Diophantine equations, but at the same time do not deviate too much from the structure of the original tree. This suggests the adoption of approximate ratios instead of exact ones, and also raises the potential to investigate the trade-off between dataset increase and tree similarity further.

The medium-term development goal is to have the proposed technique implemented as a standard data engineering service to accommodate hiding requests, coupled with an appropriate environment where one could specify the importance of each hiding request. On research and development aspects, the most pressing questions are related to the ability to handle multi-valued (also numeric) attributes and multi-class trees. From a research perspective, the most important issue relates to whether the problem can be framed in terms of (integer) constraints, so that we may be able to turn to the repertoire of constraint satisfaction techniques, and to whether one can devise metrics of tree similarity (syntactic or semantic) that utilize the locality of the operations employed by the proposed method. The apparent rise in interest of privacy-preserving solutions suggests that systems with theoretical backing can be expected to appear increasingly often.

## Appendix A

**Lemma** **1.**
*The entropy of a node only depends on the ratio of its instance classes.*

**Proof.** Let E be the entropy of a node with p positive and n negative instances, with p:n=a. We assume that a≥1. $p_{i}:$ The probability of class i

$$E=-\sum^{\text{​}}p_{i}\log_{2}p_{i}=-\frac{n}{p+n}\log_{2}\frac{n}{p+n}-\frac{p}{p+n}\log_{2}\frac{p}{p+n}=$$

$$\overset{p=\mathrm{an}}{\overbrace{=}}-\frac{n}{\mathrm{an}+n}\log_{2}\frac{n}{\mathrm{an}+n}-\frac{\mathrm{an}}{\mathrm{an}+n}\log_{2}\frac{\mathrm{an}}{\mathrm{an}+n}=$$

$$-\frac{1}{a+1}\log_{2}\frac{1}{a+1}-\frac{a}{a+1}\log_{2}\frac{a}{a+1}=$$

$$\frac{1}{a+1}\log_{2}\left(a+1\right)-\frac{a}{a+1}\left(\log_{2}a-\log_{2}\left(a+1\right)\right)=$$

$$\log_{2}\left(a+1\right)-\frac{a}{a+1}\log_{2}a\;Q.E.D.$$

 □

**Lemma** **2.**
*Distributing new class instances along only one branch maximizes information gain.*

**Proof.** Let G(i) be the function that represents the information gain after the addition of k new positive instances at a node, and the distribution of i of these nodes to the left child and of k−i to the right child.

$$G\left(i\right)=G\left(p+k,n,p_{1}+i,n_{1},p_{2}+\left(k-i\right),n_{2}\;\right),\;for\;0\leq i\leq k$$

$$G\left(i\right)=E\left(p+k,n\right)-\left[E\left(p_{1}+i,n_{1}\right)\cdot \frac{p_{1}+i+n_{1}}{p+k+n}+E\left(p_{2}+\left(k-i\right),n_{2}\right)\cdot \frac{p_{2}+k-i+n_{2}}{p+k+n}\right]=$$

$$\overset{C-Constant}{\overbrace{E\left(p+k,n\right)}}-[\left(\log\frac{p_{1}+i+n_{1}}{n_{1}}-\frac{p_{1}+i}{p_{1}+i+n_{1}}\cdot \log\frac{p_{1}+i}{n_{1}}\right)\cdot \frac{p_{1}+i+n_{1}}{p+k+n}+\left(\log\frac{p_{2}+k-i+n_{2}}{n_{2}}-\frac{p_{2}+k-i}{p_{2}+k-i+n_{2}}\cdot \log\frac{p_{2}+k-i}{n_{2}}\right)\cdot \frac{p_{2}+k-i+n_{2}}{p+k+n}]=$$

$$C-\frac{1}{p+k+n}\left[\left(p_{1}+i+n_{1}\right)\cdot \log\frac{p_{1}+i+n_{1}}{n_{1}}-\left(p_{1}+i\right)\cdot \log\frac{p_{1}+i}{n_{1}}+\left(p_{2}+k-i+n_{2}\right)\cdot \log\frac{p_{2}+k-i+n_{2}}{n_{2}}-\left(p_{2}+k-i\right)\cdot \log\frac{p_{2}+k-i}{n_{2}}\right]$$

$$\overset{change\;of\;base}{\overbrace{=}}C-\frac{1}{p+k+n}\cdot \frac{1}{ln2}\left[\left(p_{1}+i+n_{1}\right)\cdot \ln\frac{p_{1}+i+n_{1}}{n_{1}}-\left(p_{1}+i\right)\cdot \ln\frac{p_{1}+i}{n_{1}}+\left(p_{2}+k-i+n_{2}\right)\cdot \ln\frac{p_{2}+k-i+n_{2}}{n_{2}}-\left(p_{2}+k-i\right)\cdot \ln\frac{p_{2}+k-i}{n_{2}}\right]$$

Taking the first derivative of G(i) we have

$$G^{\prime }\left(i\right)=0-\frac{1}{p+k+n}\cdot \frac{1}{ln2}[\ln\frac{p_{1}+i+n_{1}}{n_{1}}+\left(p_{1}+i+n_{1}\right)\cdot \frac{n_{1}}{p_{1}+i+n_{1}}\cdot \frac{1}{n_{1}}-\ln\frac{p_{1}+i}{n_{1}}-\left(p_{1}+i\right)\cdot \frac{n_{1}}{p_{1}+i}\cdot \frac{1}{n_{1}}+\left(-1\right)\cdot \ln\frac{p_{2}+k-i+n_{2}}{n_{2}}-\left(p_{2}+k-i+n_{2}\right)\cdot \frac{n_{2}}{p_{2}+k-i+n_{2}}\cdot \frac{1}{n_{2}}-\left(-1\right)\cdot \ln\frac{p_{2}+k-i}{n_{2}}-\left(p_{2}+k-i\right)\cdot \frac{n_{2}}{p_{2}+k-i}\cdot \left(-\frac{1}{n_{2}}\right)]=$$

$$-\frac{1}{p+k+n}\left[\ln\frac{p_{1}+i+n_{1}}{n_{1}}+1-\ln\frac{p_{1}+i}{n_{1}}-1-\ln\frac{p_{2}+k-i+n_{2}}{n_{2}}-1+\ln\frac{p_{2}+k-i}{n_{2}}+1\right]=$$

$$-\frac{1}{p+k+n}\left[\ln\frac{p_{1}+i+n_{1}}{p_{1}+i}+\ln\frac{p_{2}+k-i}{p_{2}+k-i+n_{2}}\right]=$$

$$-\frac{1}{p+k+n}\left[\ln\frac{\left(p_{1}+i+n_{1}\right)\cdot \left(p_{2}+k-i\right)}{\left(p_{1}+i\right)\cdot \left(p_{2}+k-i+n_{2}\right)}\right]$$

Then we find the second derivative of G(i)

$$G^{\prime \prime }\left(i\right)=-\frac{1}{p+k+n}{\left[\ln\frac{\left(p_{1}+i+n_{1}\right)\cdot \left(p_{2}+k-i\right)}{\left(p_{1}+i\right)\cdot \left(p_{2}+k-i+n_{2}\right)}\right]}^{\prime }=$$

$$\overset{A\left(i\right)<0\;for\;every\;i\in \left[0,k\right]}{\overbrace{-\frac{1}{p+k+n}\cdot \frac{\left(p_{1}+i\right)\cdot \left(p_{2}+\overset{\geq 0}{\overbrace{k-i}}+n_{2}\right)}{\left(p_{1}+i+n_{1}\right)\cdot \left(p_{2}+\overset{\geq 0}{\overbrace{k-i}}\right)}}}\cdot {\left(\frac{\left(p_{1}+i+n_{1}\right)\cdot \left(p_{2}+k-i\right)}{\left(p_{1}+i\right)\cdot \left(p_{2}+k-i+n_{2}\right)}\right)}^{\prime }=$$

$$A\left(i\right)\cdot \frac{{\left(\left(p_{1}+i+n_{1}\right)\cdot \left(p_{2}+k-i\right)\right)}^{\prime }\cdot \left(\left(p_{1}+i\right)\cdot \left(p_{2}+k-i+n_{2}\right)\right)}{\underset{B\left(i\right)>0}{\underset{︸}{{\left(\left(p_{1}+i\right)\cdot \left(p_{2}+k-i+n_{2}\right)\right)}^{2}}}}-\frac{\left(\left(p_{1}+i+n_{1}\right)\cdot \left(p_{2}+k-i\right)\right)\cdot {\left(\left(p_{1}+i\right)\cdot \left(p_{2}+k-i+n_{2}\right)\right)}^{\prime }}{\underset{B\left(i\right)>0}{\underset{︸}{{\left(\left(p_{1}+i\right)\cdot \left(p_{2}+k-i+n_{2}\right)\right)}^{2}}}}=$$

$$\overset{C\left(i\right)<0}{\overbrace{\left(\frac{A\left(i\right)}{B\left(i\right)}\right)}}\cdot \left[\left(p_{2}+k-i-p_{1}-i-n_{1}\right)\cdot \left(p_{1}+i\right)\cdot \left(p_{2}+k-i+n_{2}\right)-\left(p_{1}+i+n_{1}\right)\cdot \left(p_{2}+k-i\right)\cdot \left(p_{2}+k-i-p_{1}-i+n_{2}\right)\right]=$$

$$C\left(i\right)\cdot \left[\left(\left(p_{2}+k-i\right)-\left(p_{1}+i+n_{1}\right)\right)\cdot \left(p_{1}+i\right)\cdot \left(p_{2}+k-i+n_{2}\right)-\left(p_{1}+i+n_{1}\right)\cdot \left(p_{2}+k-i\right)\cdot \left(\left(p_{2}+k-i+n_{2}\right)-\left(p_{1}+i\right)\right)\right]=$$

$$C\left(i\right)\cdot \left[\left(p_{2}+k-i\right)\cdot \left(p_{1}+i\right)\cdot \left(p_{2}+k-i+n_{2}\right)-\left(p_{1}+i+n_{1}\right)\cdot \left(p_{1}+i\right)\cdot \left(p_{2}+k-i+n_{2}\right)-\left(p_{2}+k-i+n_{2}\right)\cdot \left(p_{1}+i+n_{1}\right)\cdot \left(p_{2}+k-i\right)+\left(p_{1}+i\right)\cdot \left(p_{1}+i+n_{1}\right)\cdot \left(p_{2}+k-i\right)\right]=$$

$$C\left(i\right)\cdot \left[\left(p_{2}+k-i\right)\cdot \left(p_{2}+k-i+n_{2}\right)\left(p_{1}+i-p_{1}-i-n_{1}\right)+\left(p_{1}+i\right)\cdot \left(p_{1}+i+n_{1}\right)\cdot \left(p_{2}+k-i-p_{2}-k+i-n_{2}\right)\right]=$$

$$C\left(i\right)\cdot \left[\left(p_{2}+k-i\right)\cdot \left(p_{2}+k-i+n_{2}\right)\cdot \left(-n_{1}\right)+\left(p_{1}+i\right)\cdot \left(p_{1}+i+n_{1}\right)\cdot \left(-n_{2}\right)\right]=$$

$$-C\left(i\right)\cdot \left[\underset{D\left(i\right)>0}{\underset{︸}{n_{1}\cdot \left(p_{2}+\overset{\geq 0}{\overbrace{k-i}}\right)\cdot \left(p_{2}+\overset{\geq 0}{\overbrace{k-i}}+n_{2}\right)+n_{2}\cdot \left(p_{1}+i\right)\cdot \left(p_{1}+i+n_{1}\right)}}\right]=$$

−C(i)· D(i)>0, for every i∈[0,k]

So, the function G(i) is convex in the interval [0,k]. The proof of Lemma 2 is completed due to a theorem that states “A convex function on a closed bounded interval attains its maximum at one of its endpoints.” □

**Lemma** **3.**
*When serially hiding two non-sibling leaves, the number of new instances to be added to maintain the max:min ratios is larger or equal to the number of instances that would have been added if the hiding requests were handled in parallel.*

**Proof.** Let a parent node have p positive and n negative instances, with p:n = r≥ 1, and let two hiding requests, each from a different branch, propagate to that node, requiring that $p_{L},\;n_{L}$ (from the left child) and $p_{R},\;n_{R}$ (from the right child) instances be added. Now assume that to maintain the p:n ratio, we must add (parallel) $p_{X}$ or $n_{X}$ instances to parent node instead of adding first $p_{1}$ or $n_{1}$ (in order to control the change due to left child) and then $p_{2}$ or $n_{2}$ (to control the change due to right child). First, we create a Table A1 with all (32) possible cases and then we have to prove Lemma 3 for each of these cases. □

**Table A1:** All possible cases to compare parallel and l serially addition for new instances.

Parallel	Serially
$\frac{a+p_{X_{I}}}{b}\;\;\;\left(I\right)$	$\frac{a+p_{1}+p_{2}}{b}\;\;\;\left(1\right)$
	$\frac{b}{a+p_{1}+p_{2}}\;\;\;\left(2\right)$
$\frac{b}{a+p_{X_{II}}}\;\;\;\left(II\right)$	$\frac{a+p_{1}}{b+n_{2}}\;\;\;\left(3\right)$
	$\frac{b+n_{2}}{a+p_{1}}\;\;\;\left(4\right)$
$\frac{a}{b+n_{X_{III}}}\;\;\;\left(III\right)$	$\frac{a}{b+n_{1}+n_{2}}\;\;\;\left(5\right)$
	$\frac{b+n_{1}+n_{2}}{a}\;\;\;\left(6\right)$
$\frac{b+n_{X_{IV}}}{a}\;\;\;\left(IV\right)$	$\frac{a+p_{2}}{b+n_{1}}\;\;\;\left(7\right)$
	$\frac{b+n_{1}}{a+p_{2}}\;\;\;\left(8\right)$

- Case (I-1):
  $$\frac{a+p_{X_{I}}}{b}=\frac{a+p_{1}+p_{2}}{b}\Leftrightarrow p_{X_{I}}=p_{1}+p_{2}\;\;\;Q.E.D.$$
- Case (I-2):
  $$\frac{a+p_{X_{I}}}{b}=\frac{b}{a+p_{1}+p_{2}}$$
  The reason that we selected option $\left(I\right),\;i.e.,\;\frac{a+p_{X_{I}}}{b}$ is that $p_{X_{I}}$ is the minimum number of instances to add in order to maintain the ratio in the parent node.
  Therefore, $p_{X_{I}}=min\left\{p_{X_{I}},p_{X_{II}},n_{X_{III}},n_{X_{IV}}\right\}$, meaning that $p_{X_{II}}\geq p_{X_{I}}\;\left(*\right).$ If we had selected option $\left(II\right),\;i.e.,\;\frac{b}{a+p_{X_{II}}}$, we would have case (II-2).
  $$i.e.\;\;\;\frac{b}{a+p_{X_{II}}}=\frac{b}{a+p_{1}+p_{2}}\Leftrightarrow p_{X_{II}}=p_{1}+p_{2}\left(**\right)$$
  From (*), (**) above, we have $p_{1}+p_{2}\geq p_{X_{I}}\;\;\;$ Q.E.D.
- Case (I-3):
  $$\frac{a+p_{X_{I}}}{b}=\frac{a+p_{1}}{b+n_{2}}\Leftrightarrow$$
  $$\Leftrightarrow ab+an_{2}+bp_{X_{I}}+n_{2}p_{X_{I}}=ab+bp_{1}\Leftrightarrow$$
  $$\Leftrightarrow an_{2}+b\left(p_{X_{I}}-p_{1}\right)+p_{X_{I}}n_{2}=0$$
  If $p_{X_{I}}\geq p_{1}$, then this case is impossible because all terms in the left-hand side in the above equation are positive.
  If $p_{X_{I}}<p_{1}$ then we have $p_{1}+n_{2}>p_{X_{I}}\;\;\;$ Q.E.D.
- Case (I-4):
  $$\frac{a+p_{X_{I}}}{b}=\frac{b+n_{2}}{a+p_{1}}$$
  The reason that we selected option $\left(I\right),\;i.e.,\;\frac{a+p_{X_{I}}}{b}$ is that $p_{X_{I}}$ is the minimum number of instances to add in order to maintain the ratio in the parent node.
  Therefore, $p_{X_{I}}=min\left\{p_{X_{I}},p_{X_{II}},n_{X_{III}},n_{X_{IV}}\right\}$, meaning that $p_{X_{II}}\geq p_{X_{I}}\;\left(*\right).$ If we had selected option $\left(II\right),\;i.e.,\;\frac{b}{a+p_{X_{II}}}$, we would have case (II-4), for which it had been proved that
  $$p_{1}+n_{2}>p_{X_{II}}\left(**\right)$$
  From (*), (**) above, we have $p_{1}+n_{2}>p_{X_{I}}\;\;\;$ Q.E.D.
- Case (I-5):
  $$\frac{a+p_{X_{I}}}{b}=\frac{a}{b+n_{1}+n_{2}}\Leftrightarrow$$
  $$\Leftrightarrow ab+a(n_{1}+n_{2})+bp_{X_{I}}+p_{X_{I}}(n_{1}+n_{2})=ab\Leftrightarrow$$
  $$\Leftrightarrow a(n_{1}+n_{2})+bp_{X_{I}}+p_{X_{I}}(n_{1}+n_{2})=0$$
  This case is impossible because all the terms in the left-hand side in the above equation are positive.
- Case (I-6):
  $$\frac{a+p_{X_{I}}}{b}=\frac{b+n_{1}+n_{2}}{a}$$
  The reason that we selected option $\left(I\right),\;i.e.,\;\frac{a+p_{X_{I}}}{b}$ is that $p_{X_{I}}$ is the minimum number of instances to add in order to maintain the ratio in the parent node.
  Therefore, $p_{X_{I}}=min\left\{p_{X_{I}},p_{X_{II}},n_{X_{III}},n_{X_{IV}}\right\}$, meaning that $n_{X_{IV}}\geq p_{X_{I}}\;\left(*\right).$ If we had selected option $\left(IV\right),\;i.e.,\;\frac{b+n_{X_{IV}}}{a}$ we would have case (IV-6),
  $$i.e.\;\;\;\frac{b+n_{X_{IV}}}{a}\;=\frac{b+n_{1}+n_{2}}{a}\Leftrightarrow n_{X_{IV}}=n_{1}+n_{2}\left(**\right)$$
  From (*), (**) above, we have $n_{1}+n_{2}>p_{X_{I}}\;\;\;$ Q.E.D.
- Case (I-7):
  $$\frac{a+p_{X_{I}}}{b}=\frac{a+p_{2}}{b+n_{1}}\Leftrightarrow$$
  $$\Leftrightarrow ab+an_{1}+bp_{X_{I}}+n_{1}p_{X_{I}}=ab+bp_{2}\Leftrightarrow$$
  $$\Leftrightarrow an_{1}+b\left(p_{X_{I}}-p_{2}\right)+n_{1}p_{X_{I}}=0$$
  If $p_{X_{I}}\geq p_{2}$ then this case is impossible, since the left-hand side in the above equation is positive.
  If $p_{X_{I}}<p_{2}$ then we have $p_{2}+n_{1}>p_{X_{I}}\;\;\;$ Q.E.D.
- Case (I-8):
  $$\frac{a+p_{X_{I}}}{b}=\frac{b+n_{1}}{a+p_{2}}$$
  The reason that we selected option $\left(I\right),\;i.e.,\;\frac{a+p_{X_{I}}}{b}$ is that $p_{X_{I}}$ is the minimum number of instances to add in order to maintain the ratio in the parent node.
  Therefore, $p_{X_{I}}=min\left\{p_{X_{I}},p_{X_{II}},n_{X_{III}},n_{X_{IV}}\right\}$, meaning that $p_{X_{II}}\geq p_{X_{I}}\;\left(*\right).$ If we had selected option $\left(II\right),\;i.e.,\;\frac{b}{a+p_{X_{II}}}$ we would have case (II-8), for which it had been proved that
  $$n_{1}+p_{2}>p_{X_{II}}\left(**\right)$$
  From (*), (**) above, we have $n_{1}+p_{2}>p_{X_{I}}\;\;\;$ Q.E.D.
- Case (II-1):
  $$\frac{b}{a+p_{X_{II}}}=\frac{a+p_{1}+p_{2}}{b}$$
  The reason that we selected option $\left(II\right),\;i.e.,\;\frac{b}{a+p_{X_{II}}}$ is that $p_{X_{II}}$ is the minimum number of instances to add in order to maintain the ratio in the parent node.
  Therefore, $p_{X_{II}}=min\left\{p_{X_{I}},p_{X_{II}},n_{X_{III}},n_{X_{IV}}\right\}$, meaning that $p_{X_{I}}\geq p_{X_{II}}\;\left(*\right).$ If we had selected option $\left(I\right),\;i.e.,\;\frac{a+p_{X_{I}}}{b}$ we would have the case (I-1), and for that it had been proven that
  $$p_{1}+p_{2}=p_{X_{I}}\left(**\right)$$
  From (*), (**) above, we have $p_{1}+p_{2}\geq p_{X_{II}}\;\;\;$ Q.E.D.
- Case (II-2):
  $$\frac{b}{a+p_{X_{II}}}=\frac{b}{a+p_{1}+p_{2}}\Leftrightarrow p_{X_{II}}=p_{1}+p_{2}\;\;\;Q.E.D.$$
- Case (II-3):
  $$\frac{b}{a+p_{X_{II}}}=\frac{a+p_{1}}{b+n_{2}}$$
  The reason that we selected option $\left(II\right),\;i.e.,\;\frac{b}{a+p_{X_{II}}}$ is that $p_{X_{II}}$ is the minimum number of instances to add in order to maintain the ratio in the parent node.
  Therefore, $p_{X_{II}}=min\left\{p_{X_{I}},p_{X_{II}},n_{X_{III}},n_{X_{IV}}\right\}$, meaning that $p_{X_{I}}\geq p_{X_{II}}\;\left(*\right).$ If we had selected option $\left(I\right),\;i.e.,\;\frac{a+p_{X_{I}}}{b}$ we would have the case (I-3), and for that it had been proved that
  $$p_{1}+n_{2}\geq p_{X_{I}}\left(**\right)$$
  From (*), (**) above, we have $p_{1}+n_{2}\geq p_{X_{II}}\;\;\;$ Q.E.D.
- Case (II-4):
  $$\frac{b}{a+p_{X_{II}}}=\frac{b+n_{2}}{a+p_{1}}\Leftrightarrow$$
  $$\Leftrightarrow ab+bp_{1}=ab+an_{2}+bp_{X_{II}}+n_{2}p_{X_{II}}\Leftrightarrow$$
  $$\Leftrightarrow an_{2}+n_{2}p_{X_{II}}+b\left(p_{X_{II}}-p_{1}\right)=0$$
  If $p_{X_{II}}\geq p_{1}$ then this case is impossible, since the left-hand side in the above equation is positive.
  If $p_{X_{II}}<p_{1}$ then we have $p_{1}+n_{2}>p_{X_{II}}\;\;\;$ Q.E.D.
- Case (II-5):
  $$\frac{b}{a+p_{X_{II}}}=\frac{a}{b+n_{1}+n_{2}}$$
  The reason that we selected option $\left(II\right),\;i.e.,\;\frac{b}{a+p_{X_{II}}}$ is that $p_{X_{II}}$ is the minimum number of instances to add in order to maintain the ratio in the parent node.
  Therefore, $p_{X_{II}}=min\left\{p_{X_{I}},p_{X_{II}},n_{X_{III}},n_{X_{IV}}\right\}$, meaning that $n_{X_{III}}\geq p_{X_{II}}\;\left(*\right).$ If we had selected option $\left(III\right),\;i.e.,\;\frac{a}{b+n_{X_{III}}}$ we would have case (III-5), and for that it had been proved that
  $$n_{1}+n_{2}=n_{X_{III\;}}\left(**\right)$$
  From (*), (**) above, we have $n_{1}+n_{2}\geq p_{X_{II}}\;\;\;$ Q.E.D.
- Case (II-6):
  $$\frac{b}{a+p_{X_{II}}}=\frac{b+n_{1}+n_{2}}{a}\Leftrightarrow$$
  $$\Leftrightarrow ab=ab+a(n_{1}+n_{2})+bp_{X_{II}}+p_{X_{II}}(n_{1}+n_{2})\Leftrightarrow$$
  $$\Leftrightarrow a(n_{1}+n_{2})+bp_{X_{II}}+p_{X_{II}}(n_{1}+n_{2})=0$$
  This case is impossible because all the terms in the left-hand side in the above equation are positive.
- Case (II-7):
  $$\frac{b}{a+p_{X_{II}}}=\frac{a+p_{2}}{b+n_{1}}$$
  The reason that we selected option $\left(II\right),\;i.e.,\;\frac{b}{a+p_{X_{II}}}$ is that $p_{X_{II}}$ is the minimum number of instances to add in order to maintain the ratio in the parent node.
  Therefore, $p_{X_{II}}=min\left\{p_{X_{I}},p_{X_{II}},n_{X_{III}},n_{X_{IV}}\right\}$, meaning that $p_{X_{I}}\geq p_{X_{II}}\;\left(*\right).$ If we had selected option $\left(I\right),\;i.e.,\;\frac{a+p_{X_{I}}}{b}$ we would have the case (I-7), and for that it had been proved that
  $$p_{2}+n_{1}>p_{X_{I}}\left(**\right)$$
  From (*), (**) above, we have $p_{2}+n_{1}\geq p_{X_{II}}\;\;\;$ Q.E.D.
- Case (II-8):
  $$\frac{b}{a+p_{X_{II}}}=\frac{b+n_{1}}{a+p_{2}}\Leftrightarrow$$
  $$\Leftrightarrow ab+bp_{2}=ab+an_{1}+bp_{X_{II}}+n_{1}p_{X_{II}}\Leftrightarrow$$
  $$\Leftrightarrow an_{1}+n_{1}p_{X_{II}}+b\left(p_{X_{II}}-p_{2}\right)=0$$
  If $p_{X_{II}}\geq p_{2}$ then this case is impossible, since the left-hand side in the above equation is positive.
  If $p_{X_{II}}<p_{2}$ then we have $p_{2}+n_{1}>p_{X_{II}}\;\;\;$ Q.E.D.
- Case (III-1):
  $$\frac{a}{b+n_{X_{III}}}=\frac{a+p_{1}+p_{2}}{b}\Leftrightarrow$$
  $$\Leftrightarrow ab=ab+b(p_{1}+p_{2})+an_{X_{III}}+n_{X_{III}}(p_{1}+p_{2})\Leftrightarrow$$
  $$\Leftrightarrow b(p_{1}+p_{2})+an_{X_{III}}+n_{X_{III}}(p_{1}+p_{2})=0$$
  This case is impossible because all the terms in the left-hand side in the above equation are positive.
- Case (III-2):
  $$\frac{a}{b+n_{X_{III}}}=\frac{b}{a+p_{1}+p_{2}}$$
  The reason that we selected option $\left(III\right),\;i.e.,\;\frac{a}{b+n_{X_{III}}}$ is that $n_{X_{III}}$ is the minimum number of instances to add in order to maintain the ratio in the parent node.
  Therefore, $n_{X_{III}}=min\left\{p_{X_{I}},p_{X_{II}},n_{X_{III}},n_{X_{IV}}\right\}$, meaning that $p_{X_{II}}\geq n_{X_{III}}\;\left(*\right).$ If we had selected option $\left(II\right),\;i.e.,\;\frac{b}{a+p_{X_{II}}}$ we would have case (II-2), and for that it had been proved that
  $$p_{X_{II}}=p_{1}+p_{2}\left(**\right)$$
  From (*), (**) above, we have $p_{1}+p_{2}\geq p_{X_{II}}\;\;\;$ Q.E.D.
- Case (III-3):
  $$\frac{a}{b+n_{X_{III}}}=\frac{a+p_{1}}{b+n_{2}}\Leftrightarrow$$
  $$\Leftrightarrow ab+an_{2}=ab+bp_{1}+an_{X_{III}}+p_{1}n_{X_{III}}\Leftrightarrow$$
  $$\Leftrightarrow bp_{1}+p_{1}n_{X_{III}}+a\left(n_{X_{III}}-n_{2}\right)=0$$
  If $n_{X_{III}}\geq n_{2}$ then this case is impossible, since the left-hand side in the above equation is positive.
  If $n_{X_{III}}<n_{2}$ then we have $n_{2}+p_{1}>n_{X_{III}}\;\;\;$ Q.E.D.
- Case (III-4):
  $$\frac{a}{b+n_{X_{III}}}=\frac{b+n_{2}}{a+p_{1}}$$
  The reason that we selected option $\left(III\right),\;i.e.,\;\frac{a}{b+n_{X_{III}}}$ is that $n_{X_{III}}$ is the minimum number of instances to add in order to maintain the ratio in the parent node.
  Therefore, $n_{X_{III}}=min\left\{p_{X_{I}},p_{X_{II}},n_{X_{III}},n_{X_{IV}}\right\}$, meaning that $p_{X_{I}}\geq n_{X_{III}}\;\left(*\right).$ If we had selected option $\left(I\right),\;i.e.,\;\frac{a+p_{X_{I}}}{b}$ we would have case (I-4), and for that it had been proved that
  $$p_{1}+n_{2}>p_{X_{I}}\left(**\right)$$
  From (*), (**) above, we have $p_{1}+n_{2}\geq n_{X_{III}}\;\;\;$ Q.E.D.
- Case (III-5):
  $$\frac{a}{b+n_{X_{III}}}=\frac{a}{b+n_{1}+n_{2}}\Leftrightarrow n_{X_{III}}=n_{1}+n_{2}\;\;\;Q.E.D.$$
- Case (III-6):
  $$\frac{a}{b+n_{X_{III}}}=\frac{b+n_{1}+n_{2}}{a}$$
  The reason that we selected option $\left(III\right),\;i.e.,\;\frac{a}{b+n_{X_{III}}}$ is that $n_{X_{III}}$ is the minimum number of instances to add in order to maintain the ratio in the parent node.
  Therefore, $n_{X_{III}}=min\left\{p_{X_{I}},p_{X_{II}},n_{X_{III}},n_{X_{IV}}\right\}$ meaning that $n_{X_{IV}}\geq n_{X_{III}}\;\left(*\right).$ If we had selected option $\left(IV\right),\;i.e.,\;\frac{b+n_{X_{IV}}}{a}$ we would have the case (IV-6), and for that it had been proved that
  $$n_{1}+n_{2}=n_{X_{IV}}\left(**\right)$$
  From (*), (**) above, we have $n_{1}+n_{2}\geq n_{X_{III}}\;\;\;$ Q.E.D.
- Case (III-7):
  $$\frac{a}{b+n_{X_{III}}}=\frac{a+p_{2}}{b+n_{1}}\Leftrightarrow$$
  $$\Leftrightarrow ab+an_{1}=ab+bp_{2}+an_{X_{III}}+p_{2}n_{X_{III}}\Leftrightarrow$$
  $$\Leftrightarrow bp_{2}+p_{2}n_{X_{III}}+a\left(n_{X_{III}}-n_{1}\right)=0$$
  If $n_{X_{III}}\geq n_{1}$ then this case is impossible, since the left-hand side in the above equation is positive.
  If $n_{X_{III}}<n_{1}$ then we have $n_{1}+p_{2}>n_{X_{III}}\;\;\;$ Q.E.D.
- Case (III-8):
  $$\frac{a}{b+n_{X_{III}}}=\frac{b+n_{1}}{a+p_{2}}$$
  The reason that we selected option $\left(III\right),\;i.e.,\;\frac{a}{b+n_{X_{III}}}$, is that $n_{X_{III}}$ is the minimum number of instances to add in order to maintain the ratio in the parent node.
  Therefore, $n_{X_{III}}=min\left\{p_{X_{I}},p_{X_{II}},n_{X_{III}},n_{X_{IV}}\right\}$ meaning that $p_{X_{II}}\geq n_{X_{III}}\;\left(*\right).$ If we had selected option $\left(II\right),\;i.e.,\;\frac{b}{a+p_{X_{II}}}$ we would have case (II-8), and for that it had been proved that
  $$p_{2}+n_{1}>p_{X_{II}}\left(**\right)$$
  From (*), (**) above, we have $p_{2}+n_{1}>n_{X_{III}}\;\;\;$ Q.E.D.
- Case (IV-1):
  $$\frac{b+n_{X_{IV}}}{a}=\frac{a+p_{1}+p_{2}}{b}$$
  The reason that we selected option $\left(IV\right),\;i.e.,\;\frac{b+n_{X_{IV}}}{a}$, is that $n_{X_{IV}}$ is the minimum number of instances to add in order to maintain the ratio in the parent node.
  Therefore, $n_{X_{IV}}=min\left\{p_{X_{I}},p_{X_{II}},n_{X_{III}},n_{X_{IV}}\right\}$ meaning that $p_{X_{II}}\geq n_{X_{IV}}\;\left(*\right).$ If we had selected option $\left(II\right),\;i.e.,\;\frac{b}{a+p_{X_{II}}}$ we would have case (II-1), and for that it had been proved that
  $$p_{1}+p_{2}\geq p_{X_{II}}\left(**\right)$$
  From (*), (**) above, we have $p_{1}+p_{2}\geq n_{X_{III}}\;\;\;$ Q.E.D.
- Case (IV-2):
  $$\frac{b+n_{X_{IV}}}{a}=\frac{b}{a+p_{1}+p_{2}}\Leftrightarrow$$
  $$\Leftrightarrow ab=ab+b(p_{1}+p_{2})+an_{X_{IV}}+n_{X_{IV}}(p_{1}+p_{2})\Leftrightarrow$$
  $$\Leftrightarrow b(p_{1}+p_{2})+an_{X_{IV}}+n_{X_{IV}}(p_{1}+p_{2})=0$$
  This case is impossible because all the terms in the left-hand side in the above equation are positive.
- Case (IV-3):
  $$\frac{b+n_{X_{IV}}}{a}=\frac{a+p_{1}}{b+n_{2}}$$
  The reason that we selected option $\left(IV\right),\;i.e.,\;\frac{b+n_{X_{IV}}}{a}$ is that $n_{X_{IV}}$ is the minimum number of instances to add in order to maintain the ratio in the parent node.
  Therefore,$n_{X_{IV}}=min\left\{p_{X_{I}},p_{X_{II}},n_{X_{III}},n_{X_{IV}}\right\}$, meaning that $p_{X_{I}}\geq n_{X_{IV}}\;\left(*\right).$ If we had selected option $\left(I\right),\;i.e.,\;\frac{a+p_{X_{I}}}{b}$ we would have case (I-3), and for that it had been proved that
  $$p_{1}+n_{2}>p_{X_{I}}\left(**\right)$$
  From (*), (**) above, we have $p_{1}+n_{2}\geq n_{X_{IV}}\;\;\;$ Q.E.D.
- Case (IV-4):
  $$\frac{b+n_{X_{IV}}}{a}=\frac{b+n_{2}}{a+p_{1}}\Leftrightarrow$$
  $$\Leftrightarrow ab+an_{2}=ab+bp_{1}+an_{X_{IV}}+p_{1}n_{X_{IV}}\Leftrightarrow$$
  $$\Leftrightarrow bp_{1}+p_{1}n_{X_{IV}}+a\left(n_{X_{IV}}-n_{2}\right)=0$$
  If $n_{X_{IV}}\geq n_{2}$ then this case is impossible, since the left-hand side in the above equation is positive.
  If $n_{X_{IV}}<n_{2}$ then we have $n_{2}+p_{1}>n_{X_{IV}}\;\;\;$ Q.E.D.
- Case (IV-5):
  $$\frac{b+n_{X_{IV}}}{a}=\frac{a}{b+n_{1}+n_{2}}$$
  The reason that we selected option $\left(IV\right),\;i.e.,\;\frac{b+n_{X_{IV}}}{a}$ is that $n_{X_{IV}}$ is the minimum number of instances to add in order to maintain the ratio in the parent node.
  Therefore, $n_{X_{IV}}=min\left\{p_{X_{I}},p_{X_{II}},n_{X_{III}},n_{X_{IV}}\right\}$ meaning that $n_{X_{III}}\geq n_{X_{IV}}\;\left(*\right).$ If we had selected option $\left(III\right),\;i.e.,\;\frac{a+p_{X_{I}}}{b}$ we would have case (III-5), and for that it had been proved that
  $$n_{X_{III}}=n_{1}+n_{2}\left(**\right)$$
  From (*), (**) above, we have $n_{1}+n_{2}\geq n_{X_{IV}}\;\;\;$ Q.E.D.
- Case (IV-6):
  $$\frac{b+n_{X_{IV}}}{a}=\frac{b+n_{1}+n_{2}}{a}\Leftrightarrow n_{X_{IV}}=n_{1}+n_{2}Q.E.D.$$
- Case (IV-7):
  $$\frac{b+n_{X_{IV}}}{a}=\frac{a+p_{2}}{b+n_{1}}$$
  The reason that we selected option $\left(IV\right),\;i.e.,\;\frac{b+n_{X_{IV}}}{a}$ is that $n_{X_{IV}}$ is the minimum number of instances to add in order to maintain the ratio in the parent node.
  Therefore, $n_{X_{IV}}=min\left\{p_{X_{I}},p_{X_{II}},n_{X_{III}},n_{X_{IV}}\right\}$, meaning that $p_{X_{I}}\geq n_{X_{IV}}\;\left(*\right).$ If we had selected option $\left(I\right),\;i.e.,\;\frac{a+p_{X_{I}}}{b}$ we would have case (I-7), and for that it had been proven that
  $$p_{2}+n_{1}>p_{X_{I}}\left(**\right)$$
  From (*), (**) above, we have $p_{2}+n_{1}>p_{X_{I}}\geq n_{X_{IV}}\;\;\;$ Q.E.D.
- Case (IV-8):
  $$\frac{b+n_{X_{IV}}}{a}=\frac{b+n_{1}}{a+p_{2}}\Leftrightarrow$$
  $$\Leftrightarrow ab+an_{1}=ab+bp_{2}+an_{X_{IV}}+p_{2}n_{X_{IV}}\Leftrightarrow$$
  $$\Leftrightarrow bp_{2}+p_{2}n_{X_{IV}}+a\left(n_{X_{IV}}-n_{1}\right)=0$$
  If $n_{X_{IV}}\geq n_{1}$ then this case is impossible, since the left-hand side in the above equation is positive.
  If $n_{X_{IV}}<n_{1}$ then we have $n_{1}+p_{2}>n_{X_{IV}}\;\;\;$ Q.E.D.

## Appendix B

An algorithm that handles, in parallel, n hiding requests

We present an algorithm in this section which implements the look ahead technique by using linear Diophantine equations in the Swap-and-Add pass.

For every node N, we initialize a flag indicating what needs to be done as follows:

N.affected:= DO_NOTHING

We also initialize a hiding set with all leaves selected for hiding as follows:

For all L in hiding-set

L.affected:= HIDE

Other possible values for the affected flag are: MAKE_LEAF (if possible, for parents of leaves which will be hidden), and ADJUST_RATIO (for internal nodes on the path from the hidden lead to the root).

The algorithm traverses the decision tree in a depth-first-search fashion. It pushes nodes into the classic DFS stack, so that all nodes (including leaves to be hidden) are popped and processed before their ancestors; this allows us to propagate the need for change upwards.

process (node X)
  begin
    if (X.affected != DO_NOTHING) then
       // X has been selected for hiding
       if X.is-leaf() then
         X.parent.affected = MAKE_LEAF
       else
         // X is an internal node
         if (X.parent != null) then
           // set X’s parent to be affected
           X.parent.affected = ADJUST_RATIO       
           if (X.affected == MAKE_LEAF) then
             // if X is the parent of a leaf
             make-leaf (X)                                     
           elseif (X.affected == ADJUST_RATIO) then
             // if X is a node on the path
             adjust-ratio (X) 
           end-if
          end-if
        end-if
    end-if
  end
make-leaf (node X)
  begin
    // calculate required local changes
    $\mathrm{compute}\;(\pm p_{Χ}^{*},\mp n_{Χ}^{*})$
	// modify local instance population
    $X.\mathrm{add}\;(\pm p_{Χ}^{*},\mp n_{Χ}^{*})$
    if (X.parent != null) then
       // propagate change to parent
       $X.\mathrm{parent}.\mathrm{must}-\mathrm{add}\;(\pm p_{Χ}^{*},\mp n_{Χ}^{*})$
	end-if
    // turn X into a leaf if you can
    if (X.left.is-leaf() && X.right.is-leaf()) then
       X.left = null
       X.right = null
    end-if
  end
adjust-ratio (node Y)
  begin
    // calculate ratio to be preserved
    $\mathrm{compute}\;r_{Y}=p_{Y}:n_{Y}$ 
		// absorb changes from children
        $Y.\mathrm{add}\;(\pm p_{Y.left}^{*}\pm p_{Y.right}^{*},\mp n_{Y.left}^{*}\mp n_{Y.right}^{*})$
		if (Y.parent != null) then
          // propagate changes to parent
          $Y.\mathrm{parent}.\mathrm{must}-\mathrm{add}\;(\pm p_{Y.left}^{*}\pm p_{Y.right}^{*},\mp n_{Y.left}^{*}\mp n_{Y.right}^{*})$
	end-if
    // calculate LDE pairs
    $\mathrm{calculate}\;\mathrm{Diophantine}\;\mathrm{to}\;\mathrm{add}\;\left(p_{Y}^{m},n_{Y}^{m}\right),\;m\in \mathbb{N},\;m=1,\ldots$
		// select minimum pair to accommodate nodes below
        $\mathrm{select}\;\mathrm{from}\;\left(p_{Y}^{m},n_{Y}^{m}\right)\;\mathrm{according}\;\mathrm{to}\;\left(p_{Y.left}^{m.left},n_{Y.left}^{m.left}\right)+\left(p_{Y.right}^{m.right},n_{Y.right}^{m.right}\right)$
		$\mathrm{add}\;(p_{Y}^{m.Y},n_{Y}^{m.Y})\;\mathrm{to}\;Y.\mathrm{instances}$
    end
